# Optimization of quantitative proteomic analysis of clots generated from plasma of patients with venous thromboembolism

**DOI:** 10.1186/s12014-017-9173-x

**Published:** 2017-11-28

**Authors:** Aneta Stachowicz, Jakub Siudut, Maciej Suski, Rafał Olszanecki, Ryszard Korbut, Anetta Undas, Jacek R. Wiśniewski

**Affiliations:** 1 Chair of Pharmacology, Jagiellonian University Medical College, Kraków, Poland; 2 Institute of Cardiology, Jagiellonian University Medical College, Kraków, Poland; 3 Department of Proteomics and Signal Transduction, Max Planck Institute of Biochemistry, Am Klopferspitz 18, Martinsried, 82152 Planegg, Germany

**Keywords:** Proteomics, Clot, Fibrin, Venous thromboembolism, MED FASP

## Abstract

**Background:**

It is well known that fibrin network binds a large variety of proteins, including inhibitors and activators of fibrinolysis, which may affect clot properties, such as stability and susceptibility to fibrinolysis. Specific plasma clot composition differs between individuals and may change in disease states. However, the plasma clot proteome has not yet been in-depth analyzed, mainly due to technical difficulty related to the presence of a highly abundant protein—fibrinogen and fibrin that forms a plasma clot.

**Methods:**

The aim of our study was to optimize quantitative proteomic analysis of fibrin clots prepared ex vivo from citrated plasma of the peripheral blood drawn from patients with prior venous thromboembolism (VTE). We used a multiple enzyme digestion filter aided sample preparation, a multienzyme digestion (MED) FASP method combined with LC–MS/MS analysis performed on a Proxeon Easy-nLC System coupled to the Q Exactive HF mass spectrometer. We also evaluated the impact of peptide fractionation with pipet-tip strong anion exchange (SAX) method on the obtained results.

**Results:**

Our proteomic approach revealed 476 proteins repeatedly identified in the plasma fibrin clots from patients with VTE including extracellular vesicle-derived proteins, lipoproteins, fibrinolysis inhibitors, and proteins involved in immune responses. The MED FASP method using three different enzymes: LysC, trypsin and chymotrypsin increased the number of identified peptides and proteins and their sequence coverage as compared to a single step digestion. Peptide fractionation with a pipet-tip strong anion exchange (SAX) protocol increased the depth of proteomic analyses, but also extended the time needed for sample analysis with LC–MS/MS.

**Conclusions:**

The MED FASP method combined with a label-free quantification is an excellent proteomic approach for the analysis of fibrin clots prepared ex vivo from citrated plasma of patients with prior VTE.

**Electronic supplementary material:**

The online version of this article (10.1186/s12014-017-9173-x) contains supplementary material, which is available to authorized users.

## Background

Atherothrombotic vascular disease and venous thromboembolism (VTE) represent the leading causes of morbidity and mortality in the Western countries [1]. VTE, which encompasses deep vein thrombosis (DVT) and pulmonary embolism (PE), affects 1–3 patients per 1000 individuals per year [2, 3]. The pathogenesis of VTE is complex and multifactorial [3, 4]. It is the most common clinical manifestation of hypercoagulable states, including inherited (e.g. factor V Leiden, prothrombin G20210A mutation, deficiencies of antithrombin, protein C and protein S) or acquired (e.g. cancer, antiphospholipid syndrome, pregnancy, older age) conditions, which are associated with the higher risk of thrombus formation [5, 6].

Growing body of evidence indicates that fibrin clot properties represent a novel risk factor for arterial and venous thrombosis, especially in younger patients with events of unknown etiology. Interestingly, the prothrombotic plasma fibrin clot phenotype in patients with venous or arterial thrombosis involves  thin, highly-branched fibers, which are resistant to lysis [7, 8]. Moreover, it is well known that fibrin network binds a large variety of proteins, including fibronectin, thrombospondin, von Willebrand factor, α_2_-antiplasmin and interleukin-1 [9, 10]. Of note, binding of fibrinolysis inhibitors and activators to the clot structure may affect clot properties, such as stability and susceptibility to fibrinolysis. This specific plasma clot composition differs between individuals and may transiently change in disease states. However, the plasma clot proteome, particularly with prothrombotic phenotype associated with either inherited or acquired conditions, has not yet been in-depth analyzed, mainly due to technical difficulty related to the presence of a highly abundant protein—fibrinogen and fibrin that forms a plasma clot [11]. Fibrinogen is a plasma glycoprotein, which consists of 2 Aα-, 2 Bβ- and 2 γ-chains, and it accounts for as much as 75% of all proteins present in the blood clot [12]. Therefore, the proteomic analysis of plasma clots is challenging and until recently only a few reports have described the clot proteome [11, 13, 14]. Talens et al. using 2D gel electrophoresis combined with LC–MS/MS analysis identified 18 different non-covalently bound proteins of fibrin clots generated in vitro from plasma of healthy individuals [14]. In turn, Suski et al. applying shotgun proteomics revealed 62 proteins in fibrin clots prepared ex vivo from plasma of patients with acute myocardial infarction [13].

The filter aided sample preparation (FASP) and the multiple enzyme digestion FASP (MED FASP) are one of the most effective proteomic methods for sample preparation. They are reproducible and have high performance with increased depth of proteomic analysis and sequence coverage [15–17]. Thus, the aim of our study was to apply MED FASP and pipet-tip strong anion exchange (SAX) fractionation combined with LC–MS/MS analysis to optimize quantitative proteomic analysis of fibrin clots prepared ex vivo from citrated plasma of the peripheral blood.

## Methods

### Patients

Citrated plasma was obtained from four ambulatory patients with prior VTE episode. The diagnosis of DVT of the lower or upper limb required a positive finding of color duplex sonography. An iliac/caval DVT was defined as abnormal duplex flow patterns typical of thrombosis or an intraluminal filling defect on contrast computed tomography (CT) or magnetic resonance venography. The diagnosis of PE was based on the presence of typical symptoms and positive results of high resolution spiral CT. The Local Ethical Committee in Krakow approved the study (135/KBL/OIL/2013), and participants provided informed consent in accordance with the Declaration of Helsinki.

Blood samples (9:1 of 3.2% trisodium citrate) were centrifuged at 2560×*g* for 20 min (centrifuge 3K18 model, swing-out rotor no. 11133 (Sigma-Aldrich, Germany) at 20 °C within 30 min of collection, immediately frozen, and stored in aliquots at − 80 °C until further use.

### Plasma clot preparation and lysis

A fibrin clot was prepared using an assay by Pieters et al. [18]. Briefly, to 100 μL of citrate plasma was added 20 mmol/L calcium chloride and 1 U/mL thrombin (Merck, USA). This mixture was placed into plastic tubes, which were placed into a wet chamber. After 120 min of incubation tubes were connected to a reservoir of a buffer (0.05 mol/L Tris-HCl, 0.1 mol/L NaCl, pH 7.5), which rinsed a fibrin gel for 1 h. The clots were immediately frozen at −80 °C.

Clots were lysed in a buffer consisting of 0.1 M Tris-HCl, pH 8.0, 1% SDS and 50 mM DTT at 96 °C for 10 min.

### Protein and peptide determination

Total protein concentration in lysates and the peptide contents in the digests (see below) were assayed using a tryptophan fluorescence based WF-assay in a microtiter plate format [19]. For measurements, Corning Costar 96-well black flat bottom polystyrene plates (Sigma-Aldrich, Germany) were used.

### Multi-enzyme digestion filter aided sample preparation (MED FASP)

Sample aliquots containing 70 µg of total protein were processed using the MED FASP method [16] with modifications described recently [15]. Briefly, proteins were subsequently cleaved overnight with endoproteinase LysC (Wako, Germany), trypsin (Promega, USA) and chymotrypsin (Roche, Switzerland) for 18, 3 and 3 h, respectively. Peptide containing fractions were collected after each digestion step. The enzyme to protein ratio was 1:50. Digestions were carried out in 50 mM Tris-HCl for LysC and trypsin and in 50 mM Tris-HCl, pH 7.8 and 10 mM CaCl_2_ for chymotrypsin. All reactions were conducted at 37 °C. Aliquots containing 10 µg of total peptides were desalted on C_18_-StageTips [20] and concentrated to a volume of ~ 5 µL and were stored frozen at − 20 °C until mass spectrometric analysis.

### Peptide fractionation by SAX

Peptides were fractionated according to the previously described pipet-tip strong anion exchange (SAX) protocol [21] with minor modifications. Briefly, peptides were loaded into tip columns made by stacking six layers of a3M Empore anion exchange disk (1214-5012, Varian, USA) into a 200 μL micropipet tip. For column equilibration and elution of fractions, we used Britton and Robinson universal buffer (BRUB) composed of 20 mM acetic acid, 20 mM phosphoric acid and 20 mM boric acid titrated with NaOH to the desired pH. In the experiments where LysC was used, peptides were loaded into the pipet-tip column at pH 12 and the three fractions were eluted at pH 6, and 2. Tryptic peptides were loaded at pH 5 and eluted at pH 2. The flow-through and the fractions were analyzed. The peptides resulting from chymotryptic digestion were analyzed directly by liquid chromatography LC–MS/MS.

### Liquid chromatography: tandem mass spectrometry

Analysis of peptide mixtures was performed using a QExactive HF mass spectrometer (Thermo-Fisher Scientific, USA). Aliquots containing 5 µg of total peptides were chromatographed on a 50 cm column with 75 µm inner diameter packed C18material (Dr. Maisch GmbH, Germany). Peptide separation was carried out at 300 nL/min for 90 min using a two-step acetonitrile gradient 5–60% over the first 75 min and 60–95% for the following 15 min. The temperature of the column oven was 60 °C. The mass spectrometer operated in data-dependent mode with survey scans acquired at a resolution of 50 000 at m/z 400 (transient time 256 ms). Up to the top 15 most abundant isotope patterns with charge ≥ + 2 from the survey scan (300–1650 m/z) were selected with an isolation window of 1.6 m/z and fragmented by Higher Energy Collisional Dissociation  (HCD) with normalized collision energies of 25. The maximum ion injection times for the survey scan and the MS/MS scans were 20 and 60 ms, respectively. The ion target value for MS1 and MS2 scan modes was set to 3 × 10^6^ and 10^5^, respectively. The dynamic exclusion was 25 s and 10 ppm. The mass spectrometry data have been deposited to the ProteomeXchange Consortium via the PRIDE partner repository [22] with the dataset identifier: PXD006814.

### Data analysis

The spectra were searched using MaxQuant software. All raw files were searched together in single MaxQuant run using separate parameter options for MS-files from analyses of LysC, trypsin and chymotrypsin. A maximum of 2 missed cleavages was allowed. Carbamidomethylation of cysteines was set as a fixed modification. The minimum peptide length was specified to be 7 amino acids. The initial maximal mass tolerance in MS mode was set to 7 ppm, whereas fragment mass tolerance was set to 20 ppm for HCD data. The “matching between the runs”-option was used. The maximum false peptide and protein discovery rate was specified as 0.01. Protein abundances were calculated using the “Total Protein Approach” (TPA) method [23]. The calculations were performed in Microsoft Excel. The pathway analysis was performed by STRING (version 10) bioinformatic tool (Search Tool for the Retrieval of Interacting Genes/Proteins) to reveal functional interactions between proteins. Results were enriched in GO biological processes and GO cellular components with False Discovery Rate (FDR) < 0.05.

### Statistical analysis

Data are presented as a mean ± SEM. The equality of variance and the normality of the data were checked. The nonparametric Kruskal–Wallis test or one-way analysis of variance (ANOVA) with the Tukey’s post hoc test were used as appropriate. A value of *p* < 0.05 was considered as statistically significant. All analyses were performed using Statistica 10.0 software (StatSoft, Poland).

## Results and discussion

Effective protein extraction and digestion to peptides play a pivotal role in accurate quantitative proteomic analysis. Recently, proteomic reactor type methods, including FASP and MED FASP, have been described. In the FASP/MED FASP methods samples are completely dissolved in sodium dodecyl sulfate (SDS) and consecutively digested with up to three enzymes in an ultrafiltration unit [16, 17]. Here, we have performed the MED FASP method using three different enzymes: LysC, trypsin and chymotrypsin or peptide fractionation by the  SAX protocol to optimize the quantitative proteomic analysis of fibrin clots prepared ex vivo from citrated plasma of the peripheral blood drawn from patients with VTE. Plasma clots of 4 patients (1 man and 3 women) with a mean age of 36 years (aged from 30 to 58 years) were studied. There were 2 patients with isolated DVT and 2 with isolated PE. Unprovoked VTE occurred in 2 individuals. Mean time since the VTE event was 12 months.

**Fig. 1:**
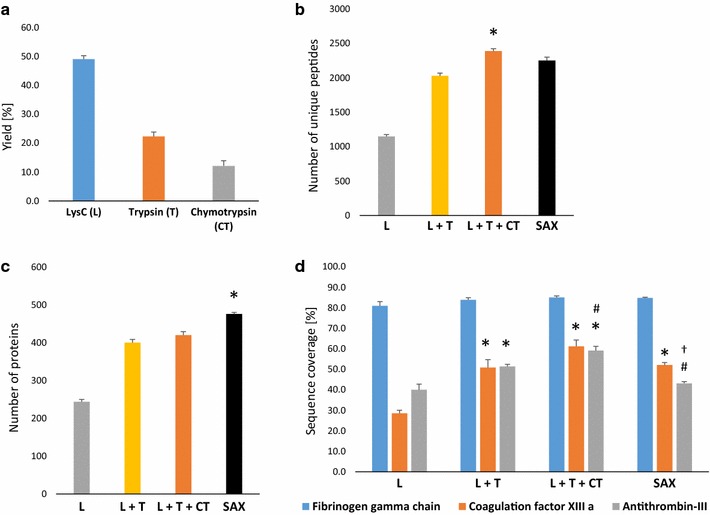
Efficiency of protein to peptide conversion after digestion with LysC, trypsin and chymotrypsin. The yields were expressed as a ratio of the amount of eluted peptides to total protein amount processed (**a**). The number of unique peptides identified after sequential digestion with LysC (L), LysC + trypsin (L + T), LysC + trypsin + chymotrypsin (L + T + CT) or peptide fractionation by pipet-tip strong anion exchange (SAX) protocol (**b**). The number of proteins identified after sequential digestion with L, L + T, L + T + CT or peptide fractionation by SAX (**c**). Sequence coverage obtained after sequential digestion with L, L + T, L + T + CT or peptide fractionation by SAX for three different proteins: fibrinogen gamma chain, coagulation factor XIII a and antithrombin-III (**d**). *p < 0.05 as compared to L, ^#^ *p* < 0.05 as compared to L + T, ^†^ *p* < 0.05 as compared to L + T + CT; mean ± SEM; n = 4 per group

Firstly, we have evaluated the efficiency of digestion with LysC, trypsin and chymotrypsin. The amount of eluted peptides to total protein amount processed was expressed as a yield (Fig. 1a). The yield of digestion with LysC, trypsin and chymotrypsin was around 50, 20 and 10%, respectively, which gave in a total 80% yield with MED FASP. The consecutive digestion of plasma fibrin clots with LysC, trypsin and chymotrypsin resulted in the increased number of the identified unique peptides (2388 ± 66) as compared to a single (1148 ± 55) (LysC) step digestion. Peptide fractionation with SAX protocol led to the similar number of identified unique peptides as obtained by MED FASP with three different enzymes (2250 ± 99) (Fig. 1b). Moreover, both consecutive digestion with LysC, trypsin and chymotrypsin and SAX fractionation provided the largest number of identified proteins (420 ± 18 and 476 ± 8, respectively) in comparison to a single (LysC) step digestion (243 ± 13) (Fig. 1c). Of note, SAX approach led to the similar number of identified proteins as compared to MED FASP with three different enzymes, but it also extended the time needed for sample analysis with LC–MS/MS, which is a result of the increased number of runs. Consecutive digestion with LysC, trypsin and chymotrypsin also resulted in a better sequence coverage than a single (LysC) or double (LysC + trypsin) step digestion and SAX fractionation as shown for coagulation factor XIII a, antithrombin-III and fibrinogen gamma chain (Fig. 1d). Interestingly, the differences in sequence coverage between digestion with one, two or three enzymes as well as SAX approach were negligible for most abundant proteins such as fibrinogen gamma chain (Fig. 1d). Noteworthy, our results are consistent with previous one, which have evaluated the MED FASP protocols, and confirm the efficiency of the MED FASP method in proteomic sample preparation [15, 16].

**Fig. 2:**
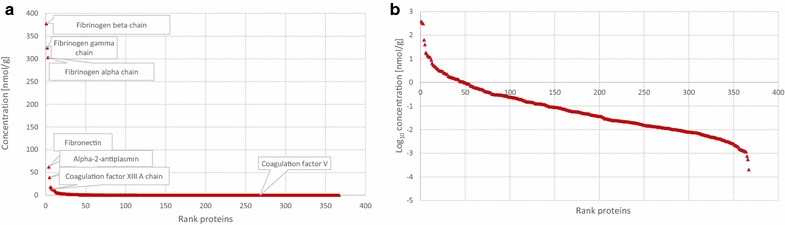
Concentrations of proteins indentified after sequential digestion with LysC + trypsin + chymotrypsin (L + T + CT) calculated by Total Protein Approach (TPA) (**a**). Log_10_ of protein concentrations illustrates the depth of proteomic analysis using MED FASP (**b**)

Recently, a protein quantification method called Total Protein Approach (TPA) has been described [23]. TPA is applicable to large scale proteomic studies and allows to calculate the protein concentrations without any requirements of spike-in standards or biochemical determination of the sample size. Protein concentrations are directly derived from the MS intensity outputs [24]. Here, we used TPA method to calculate the average concentration of proteins isolated from plasma fibrin clots prepared ex vivo from patients with VTE (Additional file 1: Table 1 and Additional file 2: Table 2). The protein concentrations of some commonly encountered proteins in human plasma fibrin clots measured by MED FASP (LysC + Trypsin + Chymotrypsin) method combined with LC–MS/MS were shown in Table 1. Clearly, the most abundant protein in human plasma clots was fibrinogen. Our results indicate that the concentrations of their three chains were almost equal (around 300 nmol/g) and were at least 10 times higher than concentrations of other abundant proteins, such as alpha-2-antiplasmin or alpha-2-macroglobulin (Fig. 2a). In turn, coagulation factors, e.g. coagulation factor IX, XII or V were present in human plasma clots at very low concentrations around 1–20 pmol/g (Table 1). Moreover, the depth of our proteomic analysis using MED FASP was around six order of magnitude, which was illustrated by log_10_ of protein concentrations shown in Fig. 2b. It indicates that MED FASP method combined with a label-free quantification is an excellent proteomic approach for the analysis of fibrin clots prepared ex vivo from plasma of patients with VTE.

**Table 1:** The average concentration of some selected proteins present in human plasma fibrin clots measured by MED FASP (LysC + Typsin + Chymotrypsin) method combined with LC–MS/MS and Total Protein Approach (TPA)

Protein IDs	Protein names	Gene names	Average concentration (nmol/g)
P08697	Alpha-2-antiplasmin	SERPINF2	39.2436
P01023	Alpha-2-macroglobulin	A2M	16.1977
P01008	Antithrombin-III	SERPINC1	1.6678
P02749	Beta-2-glycoprotein 1	APOH	0.1782
P00740	Coagulation factor IX	F9	0.0040
P12259	Coagulation factor V	F5	0.0125
P00451	Coagulation factor VIII	F8	0.0005
P00748	Coagulation factor XII	F12	0.0135
P00488	Coagulation factor XIII A chain	F13A1	13.5540
P05160	Coagulation factor XIII B chain	F13B	0.0347
P02671	Fibrinogen alpha chain	FGA	303.1711
P02675	Fibrinogen beta chain	FGB	377.2742
P02679	Fibrinogen gamma chain	FGG	324.3229
P02751	Fibronectin	FN1	62.3088
P00747	Plasminogen	PLG	2.8753
P00734	Prothrombin	F2	2.3480
P04004	Vitronectin	VTN	1.3471
P04275	von Willebrand factor	VWF	0.2985

**Fig. 3:**
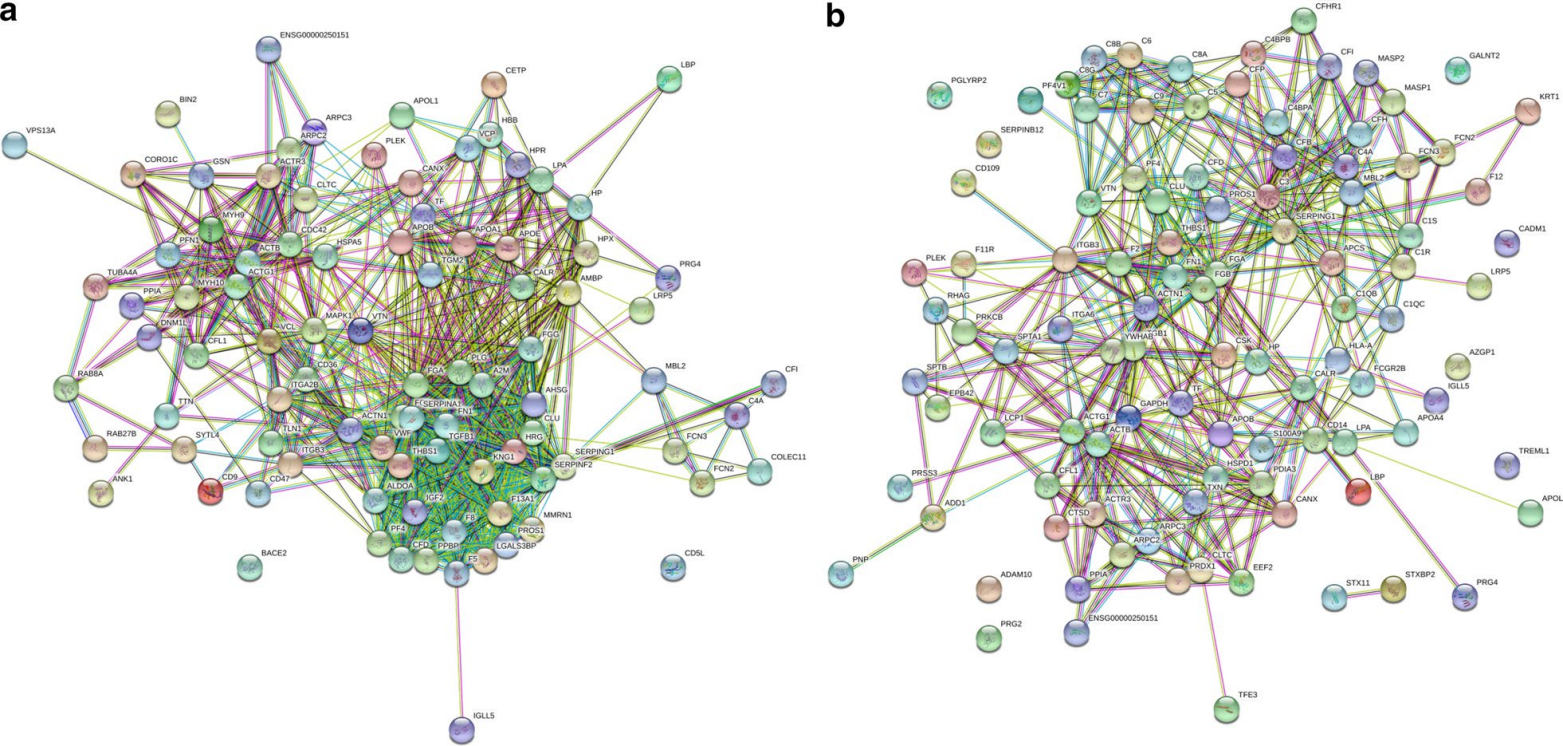
Bioinformatic analysis by STRING (version 10) of vesicle-mediated transport (**a**) and immune system (**b**) proteins identified with MED FASP (LysC + Typsin + Chymotrypsin) method combined with LC–MS/MS reveals functional interactions between proteins. Each node represents a protein and each edge represents an association. Stronger associations are represented by thicker lines

We also carried out a bioinformatic analysis of human plasma fibrin clots from patients with VTE, which were measured by MED FASP (LysC + Typsin + Chymotrypsin) method combined with LC–MS/MS. Such an analysis revealed many functional interactions between proteins, which indicates that the most of identified proteins were engaged in common molecular pathways (Fig. 3). To identify these pathways we used STRING (version 10) bioinformatic tool. Among GO biological processes significantly enriched in human plasma clots we revealed molecular pathways, which we were expecting to find, responsible for blood coagulation, platelet activation and degranulation, fibrinolysis, immune system process, regulation of blood coagulation or response to stress (Table 2). Additionally, we performed GO analysis by STRING of cellular components significantly enriched in human plasma clots from patients with VTE. Interestingly, we found that a large number of proteins identified by MED FASP method combined with LC–MS/MS are derived from various types of extracellular vesicles (EVs): exosomes, microparticles or membrane-bounded vesicles (Table 3). EVs are released to the extracellular environment by platelets, and to lesser extent by other cells, in particular endothelial cells, monocytes or neutrophils and they facilitate intercellular communication in various processes, e.g. immune response, inflammation or blood coagulation [25]. Importantly, circulating EVs could contribute to increased risk of VTE and arterial thrombosis [26, 27]. Indeed, higher levels of EVs have been observed in carriers of inherited thrombophilic states [28, 29]. The presence of EVs in human plasma fibrin clots derived from patients with VTE could confirm its role in the pathogenesis of thrombosis.

**Table 2:** The selected GO biological processes enriched in human plasma fibrin clots by STRING (version 10) bioinformatic tool

Pathway ID	Pathway description	Observed gene count	False discovery rate
GO.0072376	Protein activation cascade	46	4.39E−61
GO.0042060	Wound healing	94	6.01E−53
GO.0007596	Blood coagulation	85	1.47E−52
GO.0009611	Response to wounding	95	1.32E−50
GO.0050878	Regulation of body fluid levels	90	8.85E−50
GO.0030168	Platelet activation	61	9.95E−50
GO.0002576	Platelet degranulation	43	1.88E−48
GO.0065008	Regulation of biological quality	167	1.44E−45
GO.0006950	Response to stress	173	8.05E−42
GO.0006956	Complement activation	30	2.20E−39
GO.0016192	Vesicle-mediated transport	91	1.54E−32
GO.0001775	Cell activation	69	5.21E−32
GO.0006887	Exocytosis	49	1.78E−30
GO.2000257	Regulation of protein activation cascade	20	8.87E−28
GO.0006959	Humoral immune response	33	1.54E−25
GO.0002376	Immune system process	101	1.83E−21
GO.0030193	Regulation of blood coagulation	24	5.05E−20
GO.0030195	Negative regulation of blood coagulation	19	4.06E−19
GO.0042730	Fibrinolysis	14	1.23E−17

**Table 3:** The selected cellular components (GO term) enriched in human plasma fibrin clots by STRING (version 10) bioinformatic tool

Pathway ID	Pathway description	Observed gene count	False discovery rate
GO.1903561	Extracellular vesicle	270	6.40E−147
GO.0070062	Extracellular exosome	269	1.77E−146
GO.0031988	Membrane-bounded vesicle	280	1.98E−135
GO.0031982	Vesicle	279	8.46E−131
GO.0005576	Extracellular region	294	3.38E−123
GO.0072562	Blood microparticle	74	4.48E−102
GO.0005615	Extracellular space	164	3.89E−97
GO.0031091	Platelet alpha granule	31	1.39E−35
GO.0044433	Cytoplasmic vesicle part	54	1.81E−23
GO.0043227	Membrane-bounded organelle	286	3.17E−18
GO.0034358	Plasma lipoprotein particle	16	1.17E−16
GO.0034364	High-density lipoprotein particle	13	8.31E−15
GO.0030054	Cell junction	63	8.70E−15

## Conclusion

In this study, we have applied MED FASP and SAX fractionation to optimize quantitative proteomic analysis of fibrin clots prepared ex vivo from citrated plasma of the peripheral blood drawn from patients with prior VTE. Our proteomic approach revealed for the first time 476 proteins repeatedly identified in the plasma fibrin clots from patients with VTE. We observed that MED FASP method using three different enzymes: LysC, trypsin and chymotrypsin increased the number of identified peptides and proteins as well as their sequence coverage as compared to a single step digestion. Peptide fractionation with SAX protocol increased the depth of proteomic analysis, but also extended the time needed for sample analysis with LC–MS/MS. We found that the human plasma fibrin clots from patients with VTE contain extracellular region proteins and extracellular vesicles-derived proteins. Further studies on larger patient groups with different types of VTE are needed to better characterize protein clot composition using proteomic analysis.

In conclusion, the MED FASP method combined with a label-free quantification is an excellent proteomic approach for the analysis of fibrin clots prepared ex vivo from citrated plasma of patients with prior VTE.

## Additional files

**Additional file 1: Table** **1.** The list of proteins identified by MaxQuant software in plasma fibrin clots from patients with VTE, which were measured using MED FASP (LysC + Typsin + Chymotrypsin) method combined with LC-MS/MS and Total Protein Approach (TPA) (n = 4 per group)

**Additional file 2: Table** **2.** The list of proteins identified by MaxQuant software in plasma fibrin clots from patients with VTE, which were measured using pipet-tip strong anion exchange (SAX) protocol combined with LC-MS/MS and Total Protein Approach (TPA) (n = 4 per group)

## Abbreviations

DVT: deep vein thrombosis
EVs: extracellular vesicles
MED FASP: multiple enzyme digestion filter aided sample preparation
SAX: pipet-tip strong anion exchange
TPA: total protein approach
VTE: venous thromboembolism
